# Human iPSC-MSCs prevent steroid-resistant neutrophilic airway inflammation via modulating Th17 phenotypes

**DOI:** 10.1186/s13287-018-0897-y

**Published:** 2018-05-24

**Authors:** Shu-Bin Fang, Hong-Yu Zhang, Ai-Yun Jiang, Xing-Liang Fan, Yong-Dong Lin, Cheng-Lin Li, Cong Wang, Xiang-Ci Meng, Qing-Ling Fu

**Affiliations:** 1 Otorhinolaryngology Hospital, The First Affiliated Hospital, Sun Yat-sen University, 58 Zhongshan Road II, Guangzhou, 510080 Guangdong China; 2 Centre for Stem Cell Clinical Research and Application, The First Affiliated Hospital of Sun Yat-sen University, Guangzhou, 510080 China

**Keywords:** Dexamethasone, Immunoregulation, iPSC-MSCs, Neutrophilic airway inflammation, Type 17 helper T cells

## Abstract

**Background:**

Human induced pluripotent stem cells-derived mesenchymal stem cells (iPSC-MSCs) have been shown to be effective in Type 2 helper T cells (Th2)-dominant eosinophilic allergic airway inflammation. However, the role of iPSC-MSCs in Type 17 helper T cells (Th17)-dominant neutrophilic airway inflammation remains poorly studied. Therefore, this study was to explore the effects of iPSC-MSCs on an experimental mouse model of steroid-resistant neutrophilic airway inflammation and further determine the underlying mechanisms.

**Methods:**

A mouse model of neutrophilic airway inflammation was established using ovalbumin (OVA) and lipopolysaccharide (LPS). Human iPSC-MSCs were systemically administered, and the lungs or bronchoalveolar lavage fluids (BALF) were collected at 4 h and 48 h post-challenge. The pathology and inflammatory cell infiltration, the T helper cells, T helper cells-associated cytokines, nuclear transcription factors and possible signaling pathways were evaluated. Human CD4^+^ T cells were polarized to T helper cells and the effects of iPSC-MSCs on the differentiation of T helper cells were determined.

**Results:**

We successfully induced the mouse model of Th17 dominant neutrophilic airway inflammation. Human iPSC-MSCs but not dexamethasone significantly prevented the neutrophilic airway inflammation and decreased the levels of Th17 cells, IL-17A and p-STAT3. The mRNA levels of Gata3 and RORγt were also decreased with the treatment of iPSC-MSCs. We further confirmed the suppressive effects of iPSC-MSCs on the differentiation of human T helper cells.

**Conclusions:**

iPSC-MSCs showed therapeutic potentials in neutrophilic airway inflammation through the regulation on Th17 cells, suggesting that the iPSC-MSCs could be applied in the therapy for the asthma patients with steroid-resistant neutrophilic airway inflammation.

**Electronic supplementary material:**

The online version of this article (10.1186/s13287-018-0897-y) contains supplementary material, which is available to authorized users.

## Background

Asthma is characterized by heterogeneous upper airway inflammation in which different inflammatory cells are involved [1]. Based on the inflammatory cell profiles in induced sputum, neutrophilic asthma has been defined as a distinct phenotype from Type 2 helper T cells (Th2)-dominant eosinophilic asthma [2]. It has been reported that almost 50% asthma patients are attributable to this subgroup, in which a substantial presence of neutrophils is found in the airway [3]. Type 17 helper T cells (Th17) have been implicated in the pathogenesis of neutrophil-predominant asthma and the insensitivity to glucocorticoid in severe asthma [4–7]. After being stimulated by Th17-derived cytokines, the airway epithelial cells further release neutrophil-attracting cytokines or chemokines for the recruitment of the neutrophils [8]. Previous studies have shown that the neutrophils present in the airway were highly associated with the severity of airway inflammation [9, 10] and insensitivity to corticosteroid treatment in asthma patients [11, 12].

The steroid therapy is an important treatment for asthma patients in clinical practice. However, the patients with neutrophil-predominant asthma sometimes respond poorly to the steroid treatment even with high dosages, making it increasingly a great concern in the asthma therapies [13]. Although some advances have been made in the development of novel monoclonal antibodies for severe asthma, none of these biologics produced positive effects on asthma patients with severe neutrophilic airway inflammation [14]. Recently, some novel antagonists and inhibitors have been reported to reduce neutrophilic airway inflammation in experimental animal models of asthma [15–18]. However, chemical therapies are often associated with adverse side effects, and further studies on the safety and efficacy are required before being applied to humans. Therefore, it is evident that no effective therapies are currently available for the treatment of steroid-resistant neutrophilic airway inflammation, and the need for novel therapies has become extremely urgent.

We have successfully developed mesenchymal stem cells (MSCs) from human induced pluripotent stem cells (iPSCs) [19], and identified that human iPSC-MSCs have the potentials to modulate T cell phenotypes in human allergic rhinitis [20] and ameliorate Th2/eosinophil-dominant allergic airway inflammation in mice [21]. In addition, previous studies have shown that MSCs had exerted promising immunosuppressive effects on Th17 cells in some other immunoinflammatory diseases [22–24]. Thus, we hypothesized that iPSC-MSCs could exhibit therapeutic effects in steroid-resistant neutrophilic airway inflammation via the Th17 signaling pathway. It has been reported that murine bone marrow-derived MSCs (BM-MSCs) [25] or human umbilical cord blood-derived MSCs (UBC-MSCs) [26] suppressed neutrophilic airway inflammation. In their reports, they induced mouse models of neutrophilic airway inflammation using the fungal or viral infections as adjuvants. However, they did not report whether the models were steroid-resistant inflammation or not. Actually, exposure to environmental bacterial endotoxin has been considered a great risk factor for neutrophilic airway inflammation [3] and thus the steroid-resistant mouse model of neutrophilic airway inflammation triggered by allergen with an environment-relevant dose of lipopolysaccharide (LPS) would more closely mimic the pathogenesis of neutrophilic asthma in human [27]. We have previously reported that, compared to BM-MSCs and fetus-derived MSCs, iPSC-MSCs have a stronger immune privilege after transplantation [28]. It may attribute to a better therapeutic efficacy in an allogeneic transplantation. Currently, the effects of iPSC-MSCs on steroid-resistant neutrophilic airway inflammation and the underlying mechanisms remain to be further understood.

In the present study, we aimed to explore the effects of iPSC-MSCs on steroid-resistant neutrophilic airway inflammation triggered by allergen plus an environment-relevant dose of LPS, and evaluate the immunoregulatory function of iPSC-MSCs on T helper cells, especially the Th17 cells.

## Methods

### Animals

Female C57BL/6 mice (for neutrophil-dominant model) and Balb/c mice (for eosinophil-dominant model) (aged 6–8 weeks) were purchased from the Guangdong Medical Laboratory Animal Center (Guangzhou, China). All the animals were maintained in the specific pathogen-free environment. All the procedures performed in this study were approved by the Ethics Committee of The First Affiliated Hospital, Sun Yat-sen University.

### Preparation and identification of human iPSC-MSCs

The human iPSC-MSCs used in this study were prepared and identified as reported in our previous study [19]. Briefly, iPSCs reprogrammed from human urine-derived cells were further induced into iPSC-MSCs, which were characterized by the similar expression of general surface markers to BM-MSCs and potentials of osteogenic, chondrogenic, and adipogenic differentiation.

### Mouse model of neutrophilic airway inflammation

**Fig. 1:**
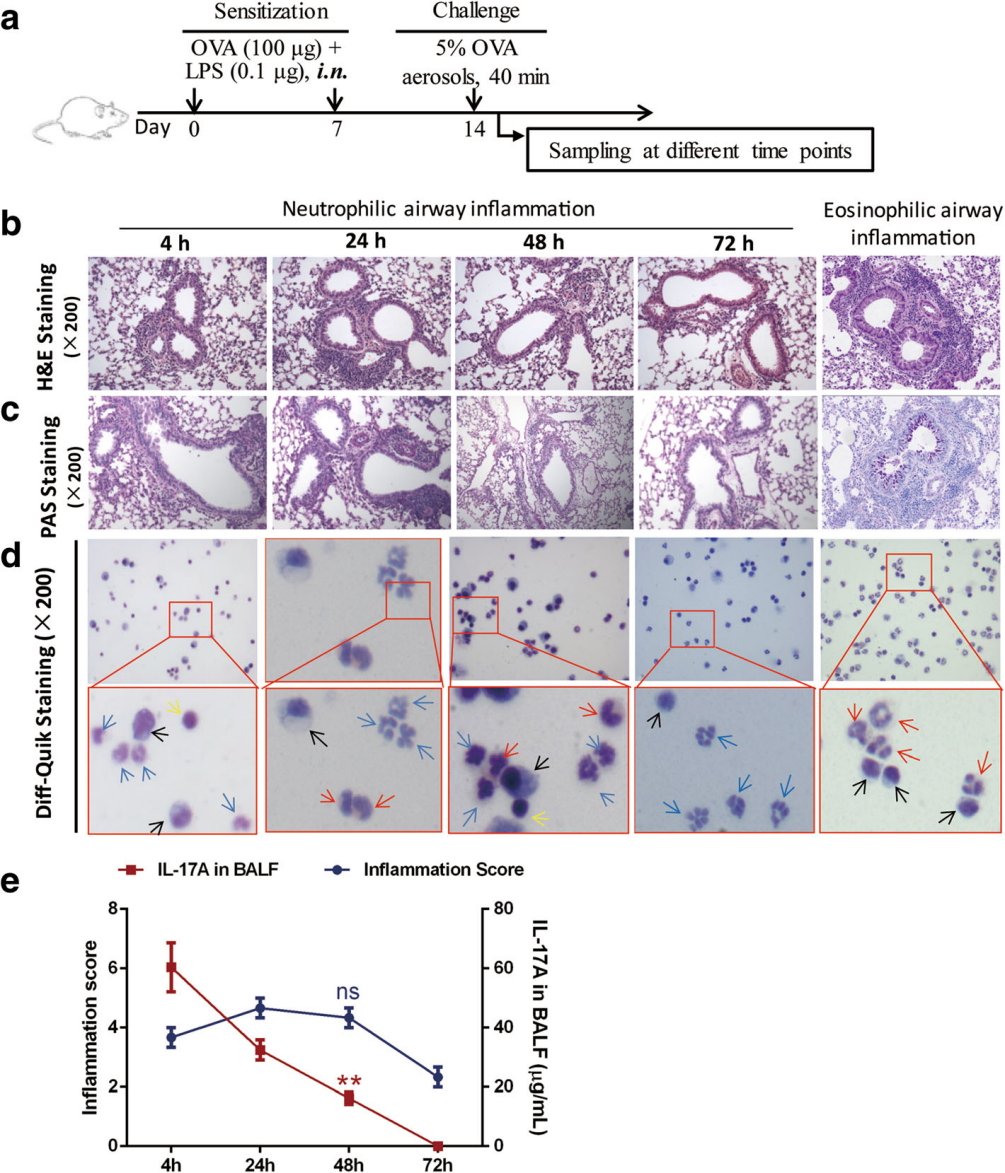
The induction of neutrophilic airway inflammation in mice. **a** Schematic diagram showing the strategy of allergen sensitization and challenge for the mouse model of neutrophilic airway inflammation. The mice were sensitized with 100 μg OVA and 0.1 μg LPS on day 0 and 7, and then were challenged with 5% OVA aerosols for 40 min on day 14. The mice were finally sacrificed at 4 h, 24 h, 48 h or 72 h post-challenge (*n* = 3). Representative H&E staining (**b**) and PAS staining (**c**) of lung tissues for neutrophilic and eosinophilic airway inflammation (× 200). Almost no PAS-positive cells were observed in the epithelial cells for neutrophilic airway inflammation while obvious PAS-positive cells were found in eosinophilic airway inflammation. **d** Representative Diff-Quik staining for inflammation cells present in BALF for neutrophilic airway inflammation and eosinophilic airway inflammation (× 200). For the neutrophilic airway inflammation, the neutrophils (*blue arrows*) but not the eosinophils (*red arrows*) were the dominant inflammatory cells in the BALF, and only a few macrophages (*black arrows*) and lymphocytes (*yellow arrows*) were observed. For the eosinophilic airway inflammation, the eosinophils were the dominant inflammatory cells in the BALF. **e** The statistical tendency of inflammation score and IL-17A level in the BALF after challenge, and the levels at 4 h and 48 h were further compared and analyzed. **P < 0.01 by *t* test. Abbreviations: *BALF* bronchoalveolar lavage fluids, *i.n*. intranasally, *LPS* lipopolysaccharide, *ns* not significant, *OVA* ovalbumin

The neutrophilic airway inflammation mouse model was developed as previously reported with minor modification [27, 29]. As shown in Fig. 1a, the mice were sensitized with 100 μg low-endotoxin Ovalbumin (OVA, *Grade V*, Sigma-Aldrich, St. Louis, MO, USA) and 0.1 μg LPS (*Escherichia coli O111:B4*, Sigma-Aldrich, St. Louis, MO, USA) in 40 μL sterile phosphate-buffered saline (PBS) on day 1 and 7 and then challenged daily with 5% aerosolized OVA for 40 min on day 14 through an air-compressing nebulizer (0.2 mL/min, Yueyue, Jiangsu, China). The negative control mice were administered with 40 μL sterile PBS and then challenged daily with PBS for 40 min on day 14. The mice were sacrificed at 4 h, 24 h, 48 h or 72 h after the challenge. Where indicated, the OVA-sensitized mice were administered with 1 × 10^6^ iPSC-MSCs (OVA/OVA/iPSC-MSC, *n* = 6 for 4 h and 48 h) intravenously or 1 mg/kg/mice dexamethasone (DEX) intraperitoneally (OVA/OVA/DEX, n = 6 for 4 h, *n* = 5 for 48 h) in 200 μL PBS on day 13 and both the negative (PBS/PBS/PBS, n = 5 for 4 h and 48 h) and positive control mice (OVA/OVA/PBS, n = 5 for 4 h and 48 h) were administered intravenously with only 200 μL PBS. The frequencies of nasal rubbing and sneezing were evaluated within 10 min after the challenge. The Th2/eosinophil-dominant airway inflammation mouse model was developed as our previous report [30, 31]. Briefly, the mice (n = 5) were sensitized with 40 μg of OVA and 4 mg of aluminum hydroxide (Thermo Fisher Scientific, Waltham, MA, USA) on days 1, 7, 14. After the administration of 200 μL PBS on day 20, the mice were further challenged with 5% OVA on days 21–25 and sacrificed at 4 h after the last challenge. After the mice were sacrificed, the bronchoalveolar lavage fluids (BALF) was collected and lung perfusion was performed to remove the remaining blood. Then the lung tissues were collected for further analysis (Left: Histopathologic analysis; Right middle lobe: PCR and western blot analysis; others: FACS analysis).

### Collection of bronchoalveolar lavage fluids (BALF)

The BALF was collected as previously reported [21]. Briefly, about 0.8 mL BALF was obtained by performing the lung lavage with 1 mL cold PBS for three times. The total cell numbers were counted with a hemocytometer and the BALF was further centrifuged at 400 g for 5 min. After the centrifugation, the supernatants were collected for the evaluation of Th1- (IFN-γ), Th2- (IL-4/13) or Th17- (IL-17A) derived cytokines (R&D Systems, Minneapolis, MN, USA). The pellets were smeared onto glass slides and stained with Diff-Quick (Baso Diagnostics Inc., Zhuhai, Guangdong, China) for differential cell counts, including neutrophils, eosinophils, lymphocytes and macrophages.

### Histopathologic evaluation of lung tissues

Lung sections were fixed with 4% paraformaldehyde for hematoxylin and eosin (H&E) staining and inflammation scores were evaluated in a blind fashion by two independent investigators based on the scoring standard as shown in Additional file 1: Table S1. Where indicated, the lung sections were also stained with Periodic acid–Schiff (PAS) for the evaluation of Goblet cell counts in airway epithelium.

### Quantitative real-time PCR

Real-time PCR was performed to detect the expression of T-bet, Gata-3 and RORγt in the lung tissues. All the primers for PCR were mouse specific. A brief description is presented in Additional file 1.

### Western blot

Western blot analysis was performed to analyze the expression of p-STAT1, p-STAT3 and p-STAT6 in the lung tissues at 4 h after challenge. The detailed information is presented in Additional file 1.

### Flow cytometry analysis of T helper cells in lung tissues

Flow cytometry analyses were performed to examine the T helper cells in lung tissues of the mouse. The detailed information is presented in Additional file 1.

### Induction of human T helper cells and co-culture with iPSC-MSCs

To investigate the effects of iPSC-MSCs on the differentiation of T helper cells, human peripheral blood mononuclear cells (PBMCs) were isolated and co-cultured with iPSC-MSCs in the presence of cytokines or antibodies for T helper cells polarization. The detailed information is presented in the Additional file 1.

### Statistical analysis

All the data were analyzed using GraphPad 6.0 (San Diego, CA, USA) and all the results were expressed as Mean ± SEM. Statistical analyses were performed using Mann-Whitney test or *t* test as indicated. A *P* value less than 0.05 were considered statistically significant.

## Results

### The neutrophilic airway inflammation elicited different responses in a time-dependent manner

To establish the mouse model of neutrophilic airway inflammation, we first explored the responses at multiple sampling time points in the development of neutrophilic airway inflammation (*n* = 3 per group). The H&E staining of the lung tissues showed that the airway inflammation in OVA-sensitized mice was observed at 4 h post-challenge. The inflammatory status continued exacerbating at 24 h, but attenuated slowly at 48 h and 72 h (Fig. 1b). However, almost no PAS-positive cells were observed in the mice with a single challenge as shown by the PAS staining of the lung tissues (Fig. 1c), suggesting that goblet cell hyperplasia in the model of neutrophilic airway inflammation was not as robust as that in the model of eosinophilic airway inflammation. Diff-Quik staining for the inflammatory cells showed that neutrophils but not eosinophils were the dominant infiltrated inflammatory cells in the airway at different sampling time points and the levels of macrophages and lymphocytes were also much lower than the neutrophils (Fig. 1d). We found many eosinophils for the Diff-Quik staining in BALF in the eosinophilic airway inflammation (Fig. 1d). Unlike the scores of airway inflammation, the levels of IL-17A in mice peaked at 4 h post-challenge and then declined sharply at 24 h, 48 h and 72 h (Fig. 1e). Therefore, it suggests that we should examine the effects of iPSC-MSCs on the airway inflammation or Th17 levels at different time points post-challenge.

### Human iPSC-MSCs ameliorated inflammatory cell infiltration in murine neutrophilic airway inflammation

**Fig. 2:**
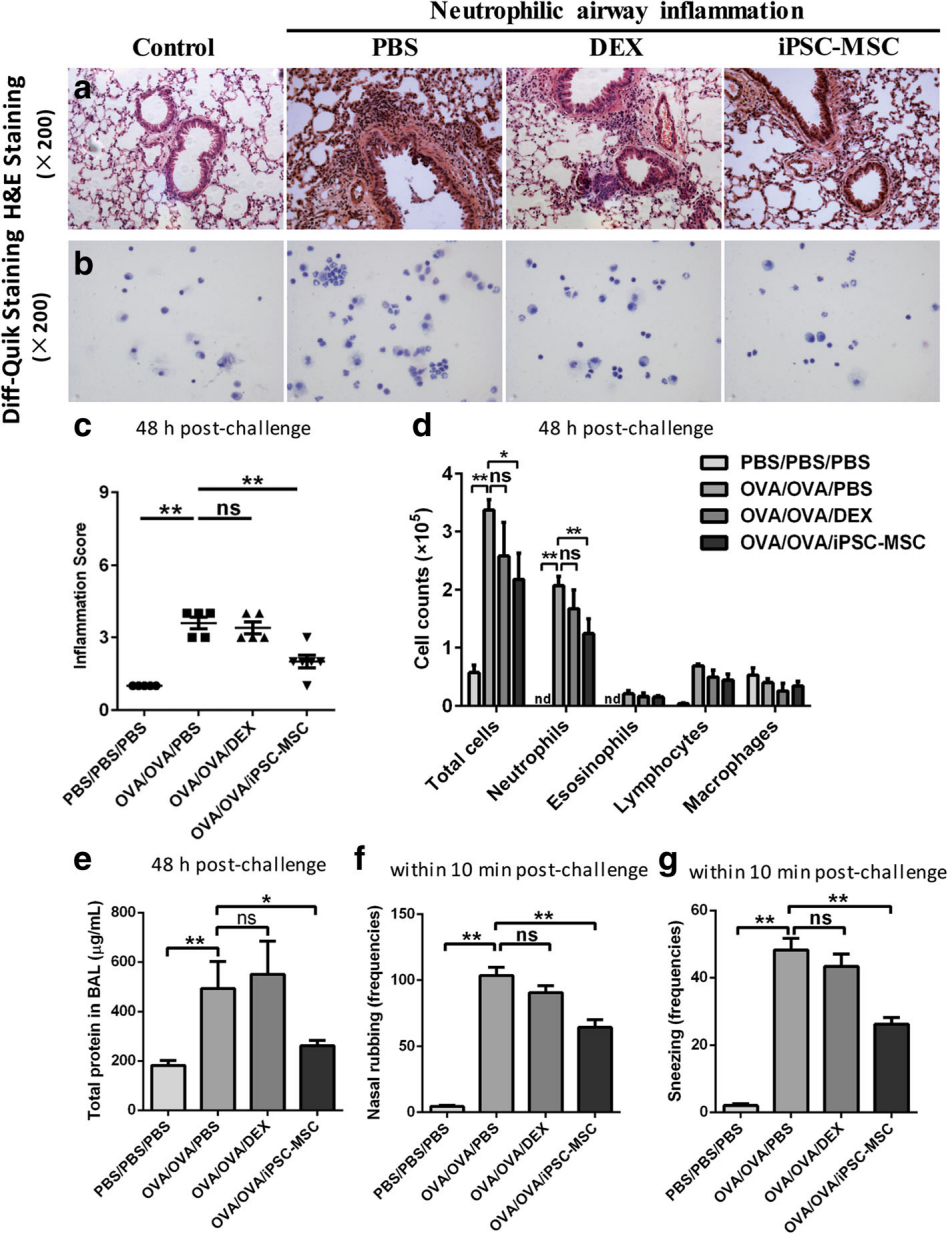
Human iPSC-MSCs reduced murine steroid-resistant airway inflammation at 48 h post-challenge. **a** Representative H&E staining of lung tissues with different treatment (× 200). Neutrophilic airway inflammation was resistant to DEX, while human iPSC-MSCs ameliorated murine airway inflammation. **b** Representative Diff-Quik staining for the inflammatory cells present in BALF with different treatment (× 200). **c** Statistical analysis of inflammatory scores. Human iPSC-MSCs but not DEX significantly decreased the inflammation score. **d** Statistical analysis of cell counts for the infiltrated inflammatory cells in BALF. Human iPSC-MSCs but not DEX significantly reduced the infiltration of inflammatory cells. **e** The levels of total protein in BALF at 48 h post-challenge. **f-g** The frequencies of nasal rubbing (**f**) and sneezing (**g**) were both significantly reduced by iPSC-MSC. *P < 0.05, **P < 0.01 by the Mann-Whitney *U* test. *Abbreviations*: *BALF* bronchoalveolar lavage fluids, *DEX* dexamethasone, *iPSC-MSCs* induced pluripotent stem cell-derived mesenchymal stem cells, *ns* not significant, *PBS* phosphate-buffered saline, *OVA* ovalbumin. *n* = 6 for OVA/OVA/MSC, *n* = 5 for the other groups

Human iPSC-MSCs were administered one day before the challenge and we evaluated the effects of iPSC-MSCs (*n* = 6) and DEX (*n* = 5) on murine histopathology for lung tissues, and the profiles of inflammatory cells in BALF at 48 h post-challenge. Obvious peribronchial inflammation was observed in the OVA/OVA/PBS mice (Fig. 2a and c, *P* < 0.01, n = 5). The treatment with DEX did not exhibit therapeutic effects on the airway inflammation. However, the airway inflammation was significantly attenuated by iPSC-MSCs (Fig. 2a and c, *P* < 0.05). Additionally, we investigated the effects of iPSC-MSCs on the profiles of inflammatory cells in BALF, in which substantial infiltration of neutrophils was found (Fig. 2b). We observed significant decreases in the numbers of total cells (*P* < 0.05) and neutrophils (*P* < 0.01) in the iPSC-MSC group, which were still poorly controlled by DEX (Fig. 2d). Also, the levels of total protein in BALF were increased in the OVA/OVA/PBS mice, and reduced by iPSC-MSCs but not DEX (Fig. 2e). All the pathogenic improvements in neutrophilic airway inflammation were consistent with the functional recovery of the frequencies of nasal rubbing (Fig. 2f) and sneezing (Fig. 2g) post-challenge with the treatment of iPSC-MSCs. These data suggest that the neutrophilic airway inflammation in the settings of the established murine model was resistant to DEX, but could be ameliorated by iPSC-MSCs.

We also examined the effects of DEX or iPSC-MSCs on airway inflammation at 4 h after the challenge. Obvious infiltration of inflammatory cells in peribronchial interstitial tissues and more total inflammatory cells, neutrophils in BALF were observed in the mice that were sacrificed at 4 h post-challenge (*P* < 0.05). However, the airway inflammation at 4 h post-challenge was not significantly decreased by DEX or iPSC-MSCs (Additional file 1: Figure S1).

### Human iPSC-MSCs inhibited Th17 levels in murine neutrophilic airway inflammation

**Fig. 3:**
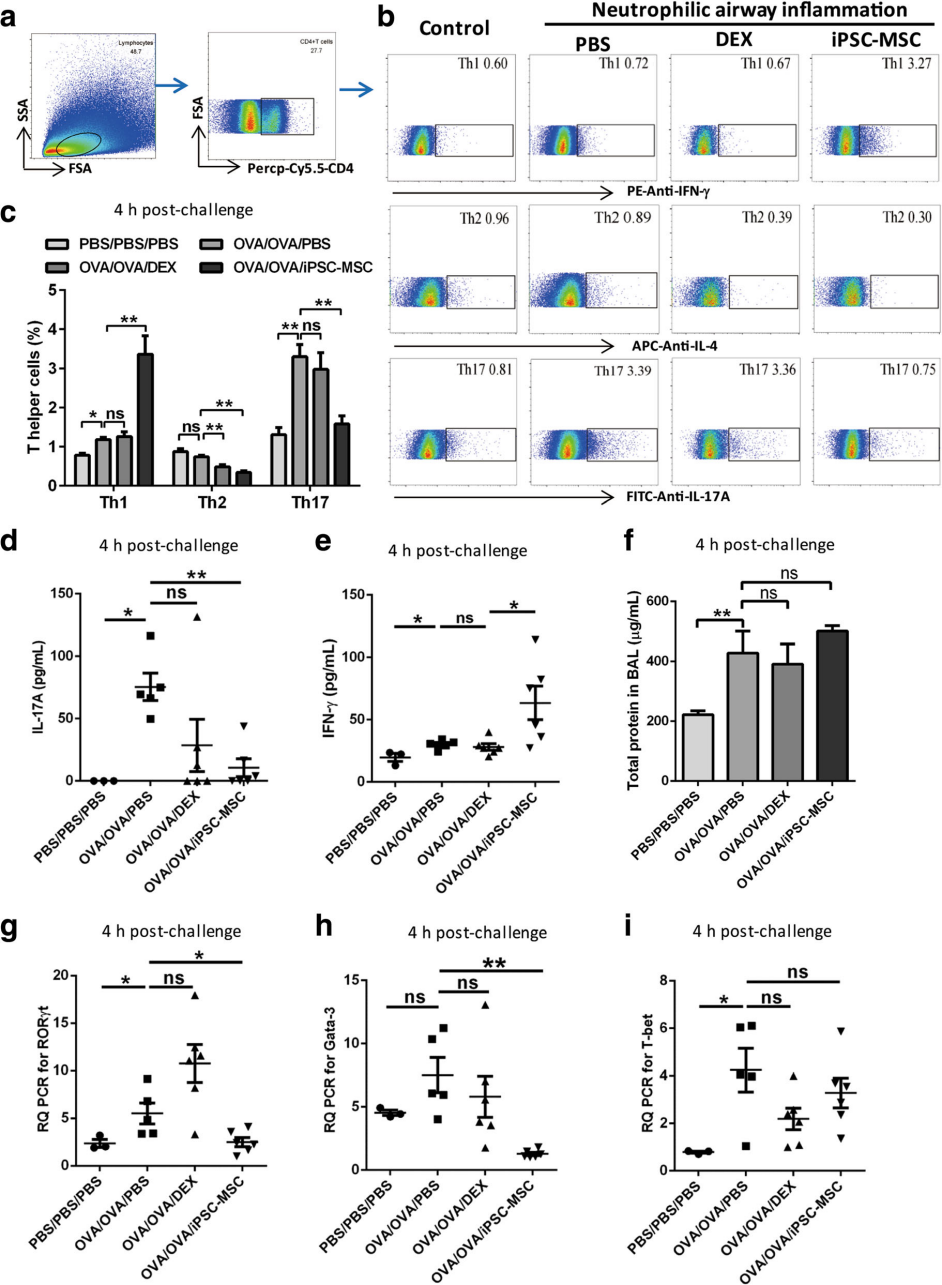
Human iPSC-MSCs inhibited Th17 level at 4 h post-challenge in a mouse model of steroid-resistant airway inflammation. **a** Representative gating strategies of flow cytometry analysis for T helper cells in mouse lung tissues. **b** Representative dot plots showing the percentages of Th1/Th2/Th17 cells in CD4^+^ T cells in different groups. **c** Statistical analysis of T helper cell percentages in lung CD4^+^ T cells. The percentage of Th17 but not Th1 and Th2 was significantly increased in neutrophilic airway inflammation. Both Th1 and Th17 were resistant to DEX and only Th2 was sensitive to DEX. iPSC-MSCs decreased both Th2 and Th17 cell levels while increased Th1 cell level in the model mouse. **d-e** Statistical analysis of IFN-γ and IL-17A levels in BALF. **f** The levels of total protein in BALF at 4 h post-challenge. **g-i** Statistical analysis of Gata-3, RORγt and T-bet levels in the lung tissues. *P < 0.05, **P < 0.01 by the Mann-Whitney *U* test. *Abbreviations*: *BALF* bronchoalveolar lavage fluids, *DEX* dexamethasone, *iPSC-MSCs* induced pluripotent stem cell-derived mesenchymal stem cells, *ns* not significant, *PBS*, phosphate-buffered saline, *OVA* ovalbumin. n = 5 for PBS/PBS/PBS and OVA/OVA/PBS, n = 6 for OVA/OVA/DEX and OVA/OVA/MSC

Th17 was reported to be involved in the neutrophilic airway inflammation [32], and our above data showed that the level of IL-17A peaked at 4 h post-challenge in our model mice. Next, we investigated the immunomodulation of iPSC-MSCs on Th cells in this neutrophilic airway inflammation model at 4 h post-challenge. The single lung cells present in lung tissues were obtained for flow cytometry analysis for Th1 (CD4^+^ IFN-γ^+^ T cells), Th2 (CD4^+^ IL-4^+^ T cells) and Th17 (CD4^+^ IL-17A^+^ T cells) (Fig. 3a and b). We observed higher Th1 (*P* < 0.05), much higher Th17 (*P* < 0.01) but not Th2 percentages in OVA/OVA/PBS mice (*n* = 5) compared to control mice (n = 5) (Fig. 3c), suggesting that Th17 was the prime T helper cells in this neutrophilic airway inflammation. Both Th2 and Th17 levels were decreased after the administration of iPSC-MSCs (*P* < 0.01, *n* = 6), while Th1 were oppositely increased (Fig. 3c, *P* < 0.01). DEX (n = 6) had no effects on Th1 and Th17 levels but slightly decreased Th2 level (Fig. 3c). Furthermore, similar tendencies to the levels of T helper cells were found for the Th1 (IFN-γ)- and Th17 (IL-17A)-derived cytokines in BALF, in which higher IL-17A was significantly decreased by iPSC-MSCs (*P* < 0.01) but not DEX while IFN-γ was significantly increased (Fig. 3d and e, *P* < 0.05). Additionally, the Th2 (IL-4/13)-derived cytokines were undetectable in all of the groups (data not shown). Both the DEX and iPSC-MSCs had no effects on the total levels of protein in BALF at 4 h post-challenge (Fig. 3f).

We further confirmed the above results by analyzing the expressions of related nuclear transcription factors and cytokines. Accordingly, the quantification for the mRNA levels of Th1 (T-bet)-, Th2 (Gata3)- and Th17 (RORγt)-associated transcription factors in lung tissues at 4 h post-challenge showed that RORγt were significantly decreased after the treatment with iPSC-MSCs (Fig. 3g, *P* < 0.05). The administration of iPSC-MSCs also decreased mRNA level of Gata3 (Fig. 3h) but had no effects on T-bet (Fig. 3i). DEX administration had no effects on the mRNA levels of T-bet, Gata3 and RORγt (Fig. 3g-i).

We also investigated the effects of iPSC-MSCs or DEX on T helper cells at 48 h post-challenge. No significant changes were observed for any of the subsets of T helper cells at 48 h post-challenge, and the treatments of both iPSC-MSCs and DEX had no effects on the T helper cells (Additional file 1: Figure S2).

These findings showed that iPSC-MSCs exhibited the immunomodulation on Th cells especially Th17 level mainly at the early stage of 4 h post-challenge. However, DEX exhibited no effects on Th1/17 cells and all the T cells-associated genes and cytokines, which further confirmed that neutrophilic airway inflammation was steroid-resistant. Taken together, these results revealed that iPSC-MSCs had the potential to inhibit the development and activity of Th17 cells in steroid-resistant airway inflammation.

### The effects of iPSC-MSCs on p-STAT3 signaling pathway in the neutrophilic airway inflammation

**Fig. 4:**
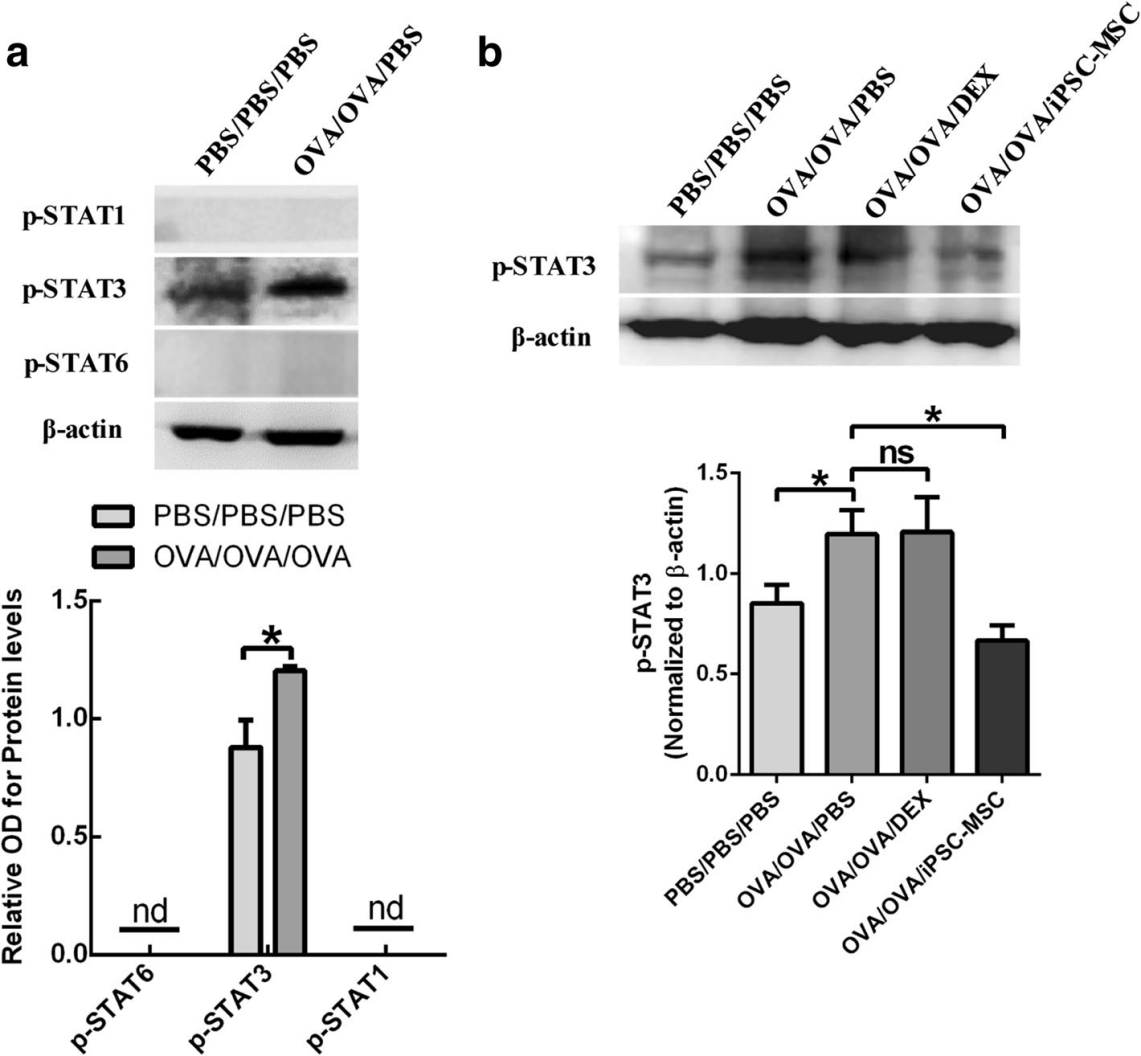
The p-STAT3 signaling was involved in the regulation of iPSC-MSCs in the mouse model of steroid-resistant airway inflammation. The lung tissues were collected at 4 h after the challenge. **a** Western blot and statistical analysis of p-STAT1, p-STAT3 and p-STAT6 expressions in the lung tissues of neutrophilic airway inflammation model. **b** Western blot analysis showed that iPSC-MSCs but not DEX significantly decreased the level of p-STAT3 in the lung tissues. *P < 0.05 by the Mann-hitney *U* test. *Abbreviations*: *DEX* dexamethasone, *iPSC-MSCs* induced pluripotent stem cell-derived mesenchymal stem cells, *ns* not significant, *nd* not detected, *PBS* phosphate-buffered saline, *OVA* ovalbumin. n = 5 for PBS/PBS/PBS and OVA/OVA/PBS, n = 6 for OVA/OVA/DEX and OVA/OVA/MSC

It was reported that STAT3 promotes the differentiation of Th17 cells [33]. To further explore the underlying mechanisms involved in the effects of iPSC-MSCs on neutrophilic airway inflammation and Th17, we determined the protein level of p-STAT3 in the mouse lungs at 4 h after the challenge. The western blot of the lung tissues showed that p-STAT3 was significantly increased after the induction of neutrophilic airway inflammation (Fig. 4a). We further identified that the level of p-STAT3 was significantly decreased after the administration of iPSC-MSCs (*n* = 6) but not DEX (n = 6) (Fig. 4b), suggesting that iPSC-MSCs may inhibit the differentiation of Th17 cells in this model of neutrophilic airway inflammation via downregulating p-STAT3 level. Additionally, we found that there was no expression of p-STAT1 and p-STAT6, which were involved in Th1, Th2 after the induction of neutrophilic airway inflammation (Fig. 4a).

### Human iPSC-MSCs inhibited the differentiation of Th cells in vitro

**Fig. 5:**
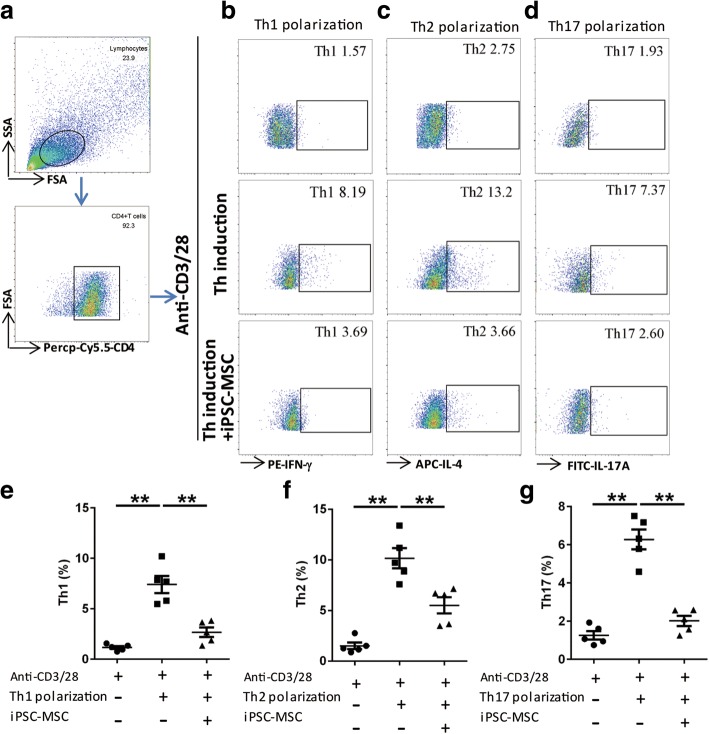
Human iPSC-MSCs inhibited the differentiation of human Th1, Th2 and Th17 cells in vitro. **a** Representative gating strategies of flow cytometry analysis for T helper cells. **b-d** Representative dot plots showing the percentages of Th1 (**b**), Th2 (**c**) and Th17 (**d**) cells in CD4^+^ T cells cultured in different T cells differentiation medium with or without iPSC-MSCs. **e-g** Statistical analysis showing that the percentages of Th1(E), Th2 (**f**) and Th17 (**g**) cells could be significantly reduced by iPSC-MSCs. **P < 0.01 by the Mann-Whitney *U* test*. Abbreviation*: *iPSC-MSCs* induced pluripotent stem cell-derived mesenchymal stem cells. n = 5 for each group

We next investigated the effects of iPSC-MSCs on the differentiation of human Th cells in vitro (*n* = 5). Purified human CD4^+^ T cells were polarized to Th1, Th2 and Th17 in different conditions respectively (Additional file 1: Table S3), activated with anti-CD3/CD28, and treated with or without human iPSC-MSCs for 5 days. Then the CD4^+^ T cells were harvested for flow cytometry analysis (Fig. 5a). We observed significant increases in the proportions for Th1 (Fig. 5b and e, *P* < 0.01), Th2 (Fig. 5c and f, *P* < 0.01) and Th17 (Fig. 5d and g, *P* < 0.01) under their polarizing conditions. The treatment with iPSC-MSCs markedly reversed the levels of all the three subsets of Th cells (Fig. 5e-g, *P* < 0.01), suggesting that iPSC-MSCs significantly suppressed the differentiation of all the three subsets of human Th cells in vitro.

## Discussion

In this study, for the first time, we demonstrated that human iPSC-MSCs inhibited the Th17 level, and further ameliorated airway inflammation in a mouse model of the steroid-resistant neutrophilic asthma. Moreover, we found that STAT3 signaling was the potential pathway involved in the immunoregulatory functions of iPSC-MSCs on neutrophilic airway inflammation. Additionally, we identified that iPSC-MSCs were capable of inhibiting the differentiation of human T helper cells in vitro.

Although the effects of the murine BM-MSCs or human UBC-MSCs in neutrophilic airway inflammation has been reported, the animal model established in this study was quite different from the two previous reports [25, 26]. In our study, LPS was used as the adjuvant to develop not only the neutrophilic but also steroid-resistant airway inflammation model. It is a good model to study the candidates for the treatment of steroid-resistant neutrophilic airway inflammation. We have previously reported that human iPSC-MSCs were effective in Th2/eosinophil-dominant asthma [21], thus we further evaluated the effects of iPSC-MSCs on steroid-resistant neutrophilic airway inflammation in our current report. MSCs derived from iPSCs, which were reprogrammed from human urine cells, were utilized in this study. For the urine cells are exfoliated renal system epithelial cells that are able to be collected under most of the circumstances except for renal failure, making it the practical and non-invasive way to collect unlimited source of human cells for reprogramming. In addition, we previously reported that the U-iPSC-MSCs exhibited obviously higher growth ability than BM-MSCs and almost no senescent cells were found even at Passage 50 [19]. These advantages enable us to provide plentiful of iPSC-MSCs in the future clinical application. We found that the administration of iPSC-MSCs prior to the challenge prevented the development of steroid-resistant neutrophilic airway inflammation, and decreased the frequencies of nasal rubbing and sneezing at 48 h post-challenge in mice. Clinically, it has been reported that the care for patients with severe asthma account for 60% of the cost of asthma even though it only makes up 3–10% of the population [34]. Among the severe asthmatics, the neutrophilic asthma is the most troublesome phenotype that is more frequently accompanied by severe symptoms and poor quality of life [35]. To our knowledge, the corticosteroid is currently the mainstay treatment for the asthmatics but fails to achieve good responses in some asthma patients with neutrophilic airway inflammation and no other effective therapies are currently available for this subpopulation [13]. Although the development of some chemicals in experimental models provided possible approaches to the therapies for this type of asthma [15, 17, 18], the general side effects of chemical treatments such as severe headache and gastrointestinal reactions should be highly concerning and could be the major obstacle in clinical application. Our findings provided us with the strong evidence that iPSC-MSCs were clinically promising in the application for the treatment of asthmatics, especially for the patients that are insensitive to steroid therapy.

As far as we know, our study was the first time to explore the effects of human iPSC-MSCs on steroid-resistant neutrophilic airway inflammation in a mouse model triggered with allergen plus an environment-relevant dose of LPS [27]. Previous studies have reported that mouse BM-MSCs or human UCB-MSCs were effective in the mouse models of neutrophilic airway inflammation, but the effects of glucocorticoid were not evaluated in these studies [25, 26]. Additionally, the models were induced with adjuvants that simulated the fungal and viral infections, which were different risk factors for neutrophilic asthma. Thus, these animal models could not possibly mimic the important feature of steroid resistance in some neutrophilic asthma patients as our model did. Also, human iPSC-MSCs have been shown to have the higher regenerative capacity and lower immunogenicity compared with BM-MSCs [36], suggesting that our iPSC-MSCs were more promising for the therapy of steroid-resistant asthma patients.

It has been demonstrated that Th17 is the major player in the pathogenesis of murine and human steroid-resistant neutrophilic asthma, in which IL-17A derived from Th17 cells further promotes the recruitment of neutrophils mainly by stimulating the production of neutrophil-attracting cytokines or chemokines from airway epithelial cells [8]. Whitehead et al. [29] previously reported that after being sensitized with allergen and increasing doses of environmentally relevant LPS, the mice that initially displayed Th2 responses gradually exhibited Th17-associated neutrophilia and they subsequently reported that 100 ng LPS was able to induce Th17-associated neutrophilia in mice [37]. Similarly, the mice were sensitized with allergen plus 0.1 μg LPS in our model and we found that the Th17 cells primed in the neutrophilic airway inflammation and was resistant to DEX treatment, suggesting that the Th17 cells should be responsible for the steroid-insensitivity of neutrophilic airway inflammation as previously reported [4]. Intriguingly, we found that the levels of Th17 cells, as well as the Th17-associated cytokine (IL-17A) and nuclear transcription factor (RORγt), were all significantly decreased by iPSC-MSCs at 4 h post-challenge, further suggesting that iPSC-MSCs exhibited the therapeutic effects on neutrophilic airway inflammation by the regulation of Th17 cells.

Interestingly, we demonstrated that human iPSC-MSCs regulated the Th17-mediated neutrophilic airway inflammation in a time-dependent manner, in which the Th17 level was decreased at 4 h post-challenge and the airway inflammation was further ameliorated at 48 h post-challenge. It suggests that the different parameters exhibited their good responses to the induction of airway inflammation and the treatment of iPSC-MSCs in different time points. We identified that iPSC-MSCs decreased the Th17 level at the early phase, and further decreased the airway inflammation at the later phase. The decrease of the high level of Th17 may be helpful to the reduction of inflammation infiltration in the later phase.

It has been demonstrated that STAT-1, STAT-6 and STAT-3 are involved in the differentiation of Th1, Th2 and Th17 respectively [33]. We observed that only p-STAT-3 but not p-STAT-1 and p-STAT-6 were expressed with high levels after the induction of steroid-resistant neutrophilic airway inflammation. Moreover, the level of p-STAT3 was significantly decreased after the administration of iPSC-MSCs. All these findings were consistent to the effects of iPSC-MSCs on the neutrophilic airway inflammation and Th17 level, which collectively suggested that iPSC-MSCs were effective in the steroid-resistant neutrophilic airway inflammation and p-STAT3 was the underlying pathway involved.

We also investigated the effects of iPSC-MSCs on the polarization of human Th cells in vitro. We found that the differentiation of Th1, Th2 and Th17 were all significantly inhibited by iPSC-MSCs. It is important that these results further confirmed the effects of iPSC-MSCs on Th17 cells. However, these findings were not totally consistent with the above in vivo experiments in which the levels of Th2 and Th17 were decreased while Th1 were reciprocally increased with the administration of iPSC-MSCs. The inconsistency could possibly be elucidated by the different activation statuses of the T cells and the different microenvironments that the iPSC-MSCs encountered between in vitro and in vivo studies.

There are some limitations in our study. First, we only focused on the effects of iPSC-MSCs on Th17 cells in our current report. It has been acknowledged that many other immune cells such as the pulmonary macrophages [38] also contribute to the steroid-insensitivity of neutrophilic airway inflammation. Therefore, further studies are required to fully explain the underlying mechanisms. Second, we only reported the prevention effects of iPSC-MSCs in our model; iPSC-MSCs were only confirmed to be effective when administered prior to OVA challenge, which somehow limits the therapeutic applicability of iPSC-MSCs. The effects of iPSC-MSCs administrated after the challenge on neutrophilic airway inflammation should be further studied in the future. Third, we used lung tissues instead of purified T cells for the qPCR and WB in our study, and we also used the PBMCs from healthy donors but not steroid-insensitive asthmatics for in vitro experiment, these could possibly lead to some unexpected results.

## Conclusions

In summary, our study showed that iPSC-MSCs were effective in steroid-insensitive neutrophilic airway inflammation. These findings emphasized that iPSC-MSCs were promising and significant alternative therapy for asthma, especially steroid-insensitive asthma.

## Additional file

Additional file 1: **Figure S1.** Human iPSC-MSCs showed no effects on murine steroid-resistant airway inflammation at 4 h post-challenge**.** (A) Representative H&E staining of lung tissues with different treatment (× 200). (B) Representative Diff-Quik staining for the inflammatory cells present in BALF with different treatment (× 200). (C) Statistical analysis of inflammatory scores for the mice that were sacrificed. No significant decreases could be observed in the mice that were treated with DEX or iPSC-MSCs. (D) Statistical analysis of cell counts for the infiltrated inflammatory cells in BALF. Neither DEX nor iPSC-MSCs could reduce the infiltration of inflammatory cells in BALF. *P < 0.05 by the Mann-Whitney *U* test. *Abbreviations*: *BALF* bronchoalveolar lavage fluids, *DEX* dexamethasone, *iPSC-MSCs* induced pluripotent stem cell-derived mesenchymal stem cells, *ns* not significant, *PBS* phosphate-buffered saline, *OVA* ovalbumin. *n* = 5 for PBS/PBS/PBS and OVA/OVA/PBS, *n* = 6 for OVA/OVA/DEX and OVA/OVA/MSC. **Figure S2.** Human iPSC-MSCs had no effects on the Th17 level at 48 h post-challenge in a mouse model of steroid-resistant airway inflammation. (A) Representative dot plots showing the percentages of Th1/Th2/Th17 cells in CD4^+^ T cells at 48 h post-challenge in murine lung tissues. (B) Statistical analysis of T helper cell percentages in lung CD4^+^ T cells at 48 h post-challenge. No significant changes of the T helper cells could be observed at 48 h post-challenge. *Abbreviations*: *DEX* dexamethasone, *iPSC-MSCs* induced pluripotent stem cell-derived mesenchymal stem cells, *ns* not significant, *PBS* phosphate-buffered solution, *OVA* ovalbumin. n = 6 for OVA/OVA/MSC, n = 5 for the other groups. (DOCX 972 kb)

## Abbreviations

BALF: Bronchoalveolar lavage fluids
BM-MSC: Bone marrow-derived mesenchymal stem cell
DEX: Dexamethasone
H&E: Hematoxylin and eosin
iPSC-MSC: Induced pluripotent stem cell-derived mesenchymal stem cell
LPS: Lipopolysaccharide
OVA: Ovalbumin
PAS: Periodic acid–Schiff
PBMCs: Peripheral blood monocytes
PBS: Phosphate-buffered saline
Th1: Type 1 helper T cells
Th17: Type 17 helper T cells
Th2: Type 2 helper T cells
UCB-MSC: Umbilical cord blood-derived MSC
