# Supplementary material for: Human iPSC-MSCs prevent steroid-resistant neutrophilic airway inflammation via modulating Th17 phenotypes
Source: Stem Cell Res Ther. 2018 May 24;9:147. doi: 10.1186/s13287-018-0897-y (PMC5968555; doi:10.1186/s13287-018-0897-y)
Supplement: Supplementary file 1 — Figure S1. Human iPSC-MSCs showed no effects on murine steroid-resistant airway inflammation at 4 h post-challenge. (A) Representative H&E staining of lung tissues with different treatment (× 200). (B) Representative Diff-Quik staining for the inflammatory cells present in BALF with different treatment (× 200). (C) Statistical analysis of inflammatory scores for the mice that were sacrificed. No significant decreases could be observed in the mice that were treated with DEX or iPSC-MSCs. (D) Statistical analysis of cell counts for the infiltrated inflammatory cells in BALF. Neither DEX nor iPSC-MSCs could reduce the infiltration of inflammatory cells in BALF. *P < 0.05 by the Mann-Whitney U test. Abbreviations: BALF bronchoalveolar lavage fluids, DEX dexamethasone, iPSC-MSCs induced pluripotent stem cell-derived mesenchymal stem cells, ns not significant, PBS phosphate-buffered saline, OVA ovalbumin. n = 5 for PBS/PBS/PBS and OVA/OVA/PBS, n = 6 for OVA/OVA/DEX and OVA/OVA/MSC. Figure S2. Human iPSC-MSCs had no effects on the Th17 level at 48 h post-challenge in a mouse model of steroid-resistant airway inflammation. (A) Representative dot plots showing the percentages of Th1/Th2/Th17 cells in CD4+ T cells at 48 h post-challenge in murine lung tissues. (B) Statistical analysis of T helper cell percentages in lung CD4+ T cells at 48 h post-challenge. No significant changes of the T helper cells could be observed at 48 h post-challenge. Abbreviations: DEX dexamethasone, iPSC-MSCs induced pluripotent stem cell-derived mesenchymal stem cells, ns not significant, PBS phosphate-buffered solution, OVA ovalbumin. n = 6 for OVA/OVA/MSC, n = 5 for the other groups. (DOCX 972 kb) [file 13287_2018_897_MOESM1_ESM.docx]

***Additional file 1***

**Human iPSC-MSCs prevent steroid-resistant neutrophilic airway inflammation via modulating Th17 phenotypes**

Shu-Bin Fang, Hong-Yu Zhang, Ai-Yun Jiang, Xing-Liang Fan, Yong-Dong Lin, Cheng-Lin Li, Cong Wang, Xiang-Ci Meng, Qing-Ling Fu

*** Correspondence:** Qing-Ling Fu: fuqingl@mail.sysu.edu.cn

**1. Additional data**

**RNA extraction and quantitative real-time PCR**

Briefly, the total RNA was extracted from murine lung tissues using Trizol reagent (Invitrogen, Paisley, UK). The complementary DNA (cDNA) was synthesized with PrimeScript RT Master Mix (Takara Bio Inc., Japan). Real-time PCR was performed using SYBR Premix ExTaq (Takara Bio Inc., Japan) to detect the expression of *T-bet*, *Gata-3* and *RORγt*. The Ct values were normalized to GADPH and the mRNA levels were calculated by the 2^-ΔΔCt^ method. The primers used in our study were shown in Table S2.

**Western blot**

The lung tissues (30-50 mg) was homogenized with ice-cold RIPA lysis buffer containing protease (Sigma, St. Louis, MO, USA) and phosphatase inhibitors (Roche Diagnostics, Mannheim, Germany) in a tissue homogenizer (Bullet Blender Storm, Next Advance, Troy, NY, USA). The lysates were then centrifuged at 300 g for 5 min and the proteins (30 μg per lane) from the supernatants were separated by SDS-PAGE and transferred to polyvinylidene difluoride membranes (Roche Diagnostics, Mannheim, Germany). The membrane was blotted with 5% BSA, washed and then incubated under gentle shaking at 4°C with primary antibodies against p-STAT1 (1:1000), p-STAT3 (1:1000), p-STAT6 (1:1000), and β-actin (1:5000). After washing, the membrane was incubated with horseradish peroxidase-conjugated anti-mouse or anti-rabbit antibody, then visualized and photographed by Enhanced Chemiluminescence Plus (Millipore Corporation, Billerica, MA, USA). The primary antibodies and secondary antibodies in this study were all purchased from Cell Signaling, Massachusetts, USA.

**Flow cytometry analysis**

The mouse lung tissues were minced and digested with 1 mg/mL collagenase type IA (Life Technologies, Carlsbad, CA, USA) and 50 μg/mL DNase I (Sigma, St. Louis, MO, USA) at 37°C for 1 h, and then the tissues were smashed against 70-µm strainers (Falcon). The digested lung cells were further suspended in high-density Percoll (ρ = 1.075 g/mL, Sigma, St. Louis, MO, USA) at a density of 10^7^/mL and overlaid with an equal volume of low-density Percoll (ρ = 1.030 g/mL, Sigma, St. Louis, MO, USA) and centrifuged at 400 g for 30 min. After the centrifugation, the single lung cells were stimulated with phorbol myristate acetate (50 ng/mL, Sigma, St. Louis, MO, USA), ionomycin (1000 ng/mL, St. Louis, Sigma, MO, USA) and GolgiStop (BD Biosciences, San Jose, CA, USA) for 5 h. The cells were first stained with anti-CD4-PerCP-Cy5.5 (Biolegend, San Diego, CA, USA) and then fixed and permeabilized for intracellular staining with anti-IFN-γ-PE, anti-IL-4-APC and anti-IL-17A-FITC (e-Bioscience, San Diego, CA, United States). After the staining, the cells were analyzed on a flow cytometer (Beckman Coulter Gallios, Fullerton, CA, USA) and the results were analyzed with FlowJo V10 (BD Biosciences, San Jose, CA, USA).

**Induction of human T helper cells and co-culture with iPSC-MSCs**

Human peripheral blood mononuclear cells (PBMCs) were separated by Ficoll-Paque PREMIUM density gradient centrifugation (1.078 g/mL, GE Healthcare, England, UK) from the buffy coats of healthy volunteers, which were provided by Guangzhou Blood Center. Then, CD4^+^ T cells were further purified from PBMCs by magnetic positive selection using the MACS CD4 microbeads (MiltenyiBiotec, Bergisch Gladbach, Germany). The purities of the CD4^+^ T cells were higher than 90% as confirmed by the flow cytometry (data not shown). To investigate the effects of iPSC-MSCs on the differentiation of T helper cells, a total number of 2×10^5^ CD4^+^ T cells were seeded in 24-well plate and co-cultured with or without 1×10^5^ iPSC-MSC in Th1, Th2 and Th17 polarizing medium as previously reported with minor modifications (Table S3) (30). Briefly, the CD4^+^ T cells were cultured in 1mL X-VIVO^TM^ 15 serum-free medium in the presence of anti-CD3/28 (1 µg/mL and 5 µg/mL, respectively) with the following cytokines (Table S3): IL-2 (10 ng/mL) and IL-12 (10 ng/mL) for Th1 polarization; IL-2 (10 ng/mL) and IL-4 (25 ng/mL) for Th2 polarization; IL-1β (10 ng/mL), IL-6 (20 ng/mL), TGF-β1 (1 ng/mL) and IL-23 (100 ng/mL) for Th17 polarization. Antibodies to IFN-γ or IL-4 were also added at a concentration of 10 μg/mL as follows: anti-IL-4 for Th1 polarization; anti-IFN-γ for Th2 polarization; anti-IFN-γ and anti-IL-4 for Th17 polarization. Anti-CD3/28, IL-2, IL-12, IL-4, IL-6, antibodies to IFN-γ and IL-4 were from BD Biosciences (San Jose, CA, USA). IL-1β, TGF-β1 and IL-23 were from RD System (Minneapolis, MN, USA). After 5 days, the T cells were collected for flow cytometry analysis. Healthy human blood buffy coats were from ‘anonymous donors’ and exemption of the written informed consent was approved by the Ethics Committee of The First Affiliated Hospital, Sun Yat-sen University, China.

**2. Additional figures and tables**

**2.1 Additional figures**

**
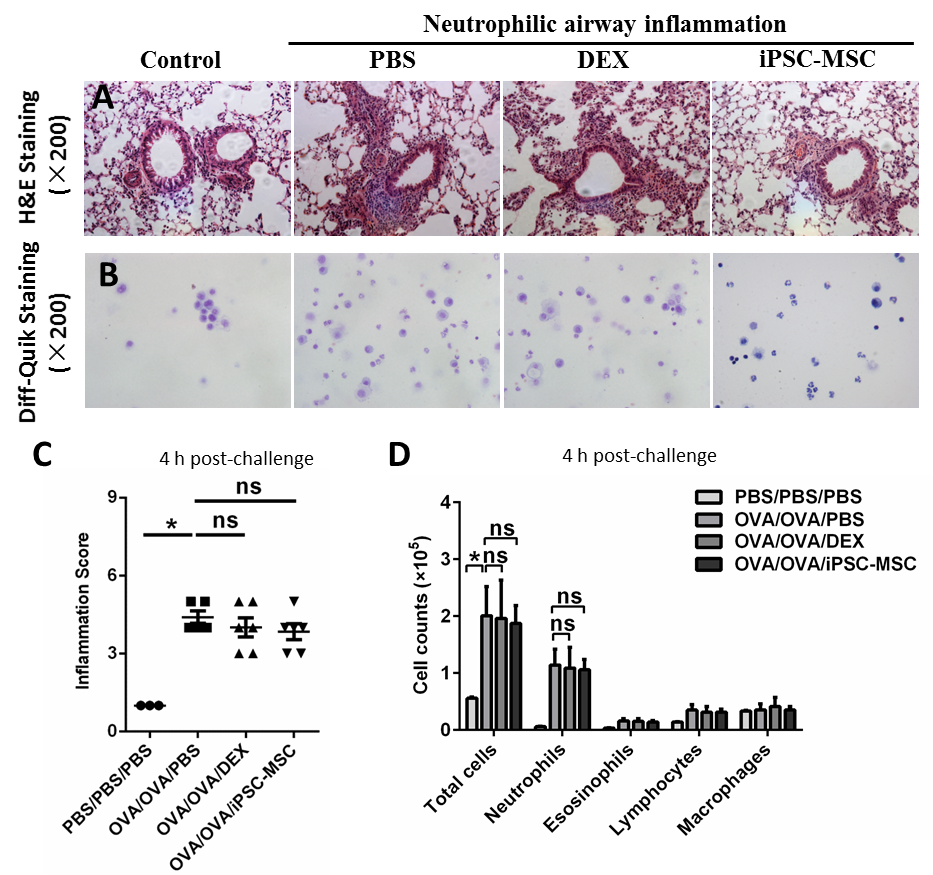
**

**Fig. S1 Human iPSC-MSCs showed no effects on murine steroid-resistant airway inflammation at 4 h post-challenge.** (A) Representative H&E staining of lung tissues with different treatment (×200). (B) Representative Diff-Quik Staining for the inflammatory cells present in BALF with different treatment (×200). (C) Statistical analysis of inflammatory scores for the mice that were sacrificed. No significant decreases could be observed in the mice that were treated with DEX or iPSC-MSCs. (D) Statistical analysis of cell counts for the infiltrated inflammatory cells in BALF. Neither DEX nor iPSC-MSCs could reduce the infiltration of inflammatory cells in BALF. **P* < 0.05 by the Mann–Whitney *U* test. Abbreviations: *DEX*, dexamethasone; *iPSC-MSCs*, induced pluripotent stem cell-derived mesenchymal stem cells; *ns,* not significant; *PBS*, phosphate buffer solution; *OVA*, ovalbumin.

**
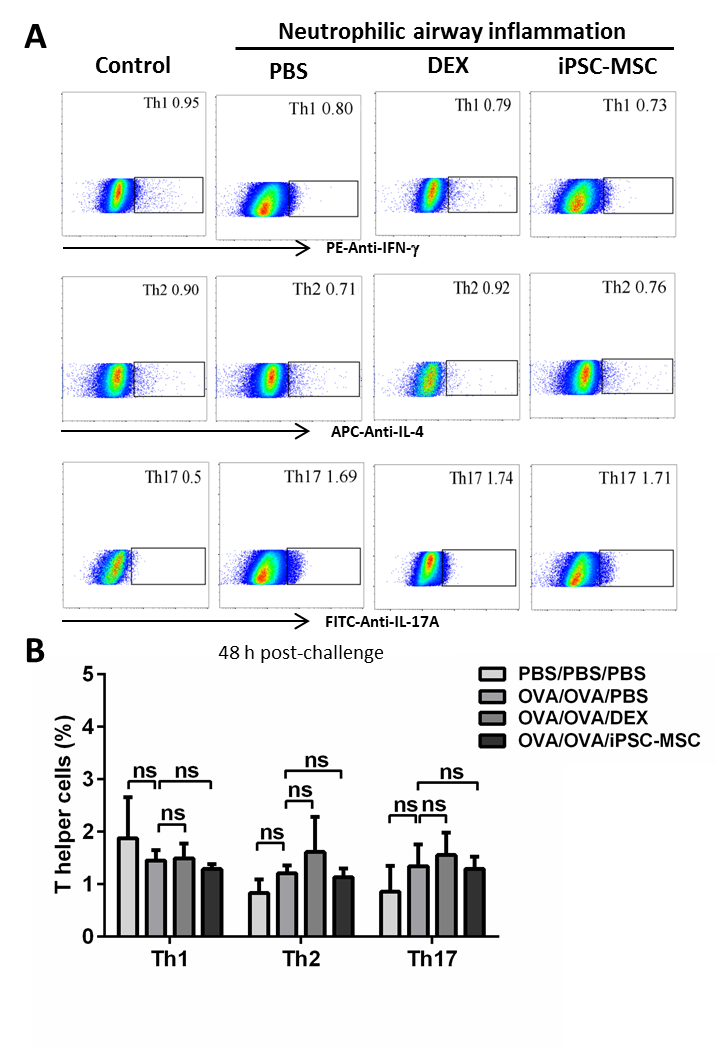
**

**Fig. S2 Human iPSC-MSCs had no effects on the Th17 level at 48 h post-challenge in a mouse model of steroid-resistant airway inflammation.** (A) Representative dot plots showing the percentages of Th1/Th2/Th17 cells in CD4^+^ T cells at 48 h post-challenge in murine lung tissues. (B) Statistical analysis of T helper cell percentages in lung CD4^+^ T cells at 48 h post-challenge. No significant changes of the T helper cells could be observed at 48 h post-challenge. Abbreviations: *DEX*, dexamethasone; *iPSC-MSCs*, induced pluripotent stem cell-derived mesenchymal stem cells; *ns*, not significant. *PBS*, phosphate buffer solution; *OVA*, ovalbumin.

**2.2 Additional tables**

**Table S1. Scores for quantifying lung inflammation.**

| **Lung inflammation** | | **Score** |
| --- | --- | --- |
| No inflammation was detectable. | | 1 |
| Bronchi were surrounded with a thin layer of inflammatory cells | few bronchi | 2 |
|  | more bronchi | 3 |
|  | most bronchi | 4 |
| Bronchi were surrounded with a thick layer of inflammatory cells | few bronchi | 5 |
|  | more bronchi | 6 |
|  | most bronchi | 7 |
| Inflammation spread into the interstitial area | severe | 8 |
|  | extreme | 9 |

**Table S2. Primers designed for quantitative real-time PCR in this study.**

| **Genes** | | **Sequences (5'to3')** |
| --- | --- | --- |
| T-bet | Sense | CTGGAGCCCACAAGCCATTA |
|  | Anti-sense | TTTCCACACTGCACCCACTT |
| Gata-3 | Sense | AAAGAAGGCATCCAGACCCG |
|  | Anti-sense | TTGAAGGAGCTGCTCTTGGG |
| RORγt | Sense | TCTACACGGCCCTGGTTCTCATCAA |
|  | Anti-sense | GGAAGGCGGCTTGGACCACGAT |

**Table S3. The details of cytokines or antibodies used for T helper cells polarization in this study.**

|  | **Cytokines or antibodies** | **Company** | **Working Concentration** |
| --- | --- | --- | --- |
| **Th1 polarization** | IL-2 | BD Biosciences | 10 ng/ml |
|  | IL-12 | BD Biosciences | 10 ng/ml |
|  | anti-IL-4 | BD Biosciences | 10 μg/mL |
| **Th2 polarization** | IL-2 | BD Biosciences | 10 ng/ml |
|  | IL-4 | BD Biosciences | 25 ng/ml |
|  | anti-IFN-γ | BD Biosciences | 10 μg/mL |
| **Th17 polarization** | IL-1β | RD System | 10 ng/ml |
|  | IL-6 | BD Biosciences | 20 ng/ml |
|  | TGF-β1 | RD System | 1 ng/ml |
|  | IL-23 | RD System | 100 ng/ml |
|  | anti-IFN-γ | BD Biosciences | 10 μg/mL |
|  | anti-IL-4 | BD Biosciences | 10 μg/mL |
